# A case of *Lomentospora prolificans* endophthalmitis treated with the novel antifungal agent Olorofim

**DOI:** 10.1186/s12348-024-00393-2

**Published:** 2024-03-22

**Authors:** Michael Dong, Fiona Pearce, Nandini Singh, Ming-Lee Lin

**Affiliations:** 1 Department of Ophthalmology, Alfred Hospital, Melbourne, Australia; 2 The Royal Victorian Eye and Ear Hospital, East Melbourne, Victoria Australia

**Keywords:** Lomentospora prolificans, Fungal endophthalmitis, Olorofim

## Abstract

**Purpose:**

To report a case of endogenous *Lomentospora prolificans* endophthalmitis treated with the novel antifungal agent Olorofim.

**Case report:**

A 57-year-old man developed disseminated *Lomentospora prolificans* with right endophthalmitis on the background of immunosuppression following lung transplantation for interstitial lung disease. He was treated with early vitrectomy, intravitreal voriconazole, and systemic Olorofim, voriconazole and terbinafine. His symptoms improved and remained stable in the right eye. Eight weeks later the patient represented with *Lomentopora prolificans* endophthalmitis in the left eye when systemic voriconazole and terbinafine treatment were withdrawn. Despite aggressive treatment he ultimately succumbed due to vascular complications of extensive disseminated disease.

**Conclusion:**

We report a rare case of disseminated *Lomentospor*o*sis* with panophthalmitis in an immunocompromised host with prolonged survival on systemic Olorofim, voriconazole and terbinafine in conjunction with pars plana vitrectomy and intravitreal voriconazole. Early suspicion of an opportunistic fungal infection is critical, as managing disseminated disease is often unsuccessful. Despite presumed inherent resistance, intravitreal and systemic voriconazole appeared to limit disease progression in the right eye. The potential synergistic effects of combined antifungal therapy with orotomides warrant further investigation.

## Introduction

*Lomentospora prolificans* (formerly known as *Scedosporium prolificans*) is a rare, emerging opportunistic fungal species that primarily affects immunocompromised individuals. *L. prolificans* has intrinsic resistance to conventional antifungals and disseminated infection confers significant mortality risk. Olorofim is a novel agent that shows promise in treating fungal infections that are resistant to conventional therapies. We present a case of *L. prolificans* panophthalmitis managed with intravitreal voriconazole, systemic terbinafine, voriconazole and Olorofim.

## Case presentation

A 57-year-old man presented to the emergency department with unilateral right eye blurred vision for three days, which predominantly affected his inferior hemifield. This was preceded by one week of progressive right periorbital erythema and oedema. He had a history of hypermetropia and no other past ophthalmic history.

His medical history included multiple myeloma diagnosed 13 years prior and in remission at the time of presentation. As part of his treatment for multiple myeloma, he received allograft stem cell transplantation. He developed interstitial lung disease secondary to severe graft-versus-host disease which resulted in bilateral sequential lung transplantation three months prior to presentation.

On routine bronchoscopy two weeks post lung transplant, a bronchoalveolar lavage (BAL) specimen cultured 1+ *Lomentospora prolificans*. The patient commenced voriconazole 200mg twice daily, terbinafine 250mg twice daily and his tacrolimus dose was reduced. Two subsequent BAL specimens six- and eight-weeks post-transplant demonstrated no growth. However, he then developed mild neutropenia and acute on chronic renal failure, with a creatinine clearance of 52mL/minute. As a result of his poor renal function, prophylactic valganciclovir was dose-reduced and trimethoprim/sulfamethoxazole was ceased.

At presentation, right visual acuity was 6/24. However, this rapidly deteriorated to counting fingers within 24 hours. Left visual acuity was 6/6. Intraocular pressures were 8mmHg bilaterally. There was inferior chemosis and inferior conjunctival injection in the right eye with a clear cornea. There was 2+ anterior chamber cells in the right eye and 1+ anterior vitreous. Fundus examination revealed moderate vitreous haze with a large area of elevated retinitis encompassing the superior macula surrounded by patchy occlusive retinal vasculitis and retinal haemorrhages. The left eye was unremarkable.

An urgent anterior chamber and vitreous aspiration was performed for microscopy and culture. The vitreous was injected with ceftazidime, vancomycin, foscarnet and voriconazole prior to both vitreous and aqueous specimens culturing 1+ Lomentospora. Antifungal sensitivities were performed which demonstrated resistance to Amphotericin B, however sensitivities to 5-Flurocytosin, anidulafungun, posaconazole and voriconazole were not able to be interpreted due a lack of established clinical breakpoints in minimum inhibitory concentration. Other blood work up demonstrated a haemoglobin of 90g/L (reference range 128–175), lymphocyte count of 0.13x10^9^/L (reference range 0.90–3.30) and neutrophil count of 1.06x10^9^/L (reference range 1.90–8.00).

Systemically, the oral voriconazole dose was increased to 250mg twice a day and micafungin was commenced at 300mg loading followed by 150mg daily in addition to terbinafine 250mg twice daily.

**Fig. 1:**
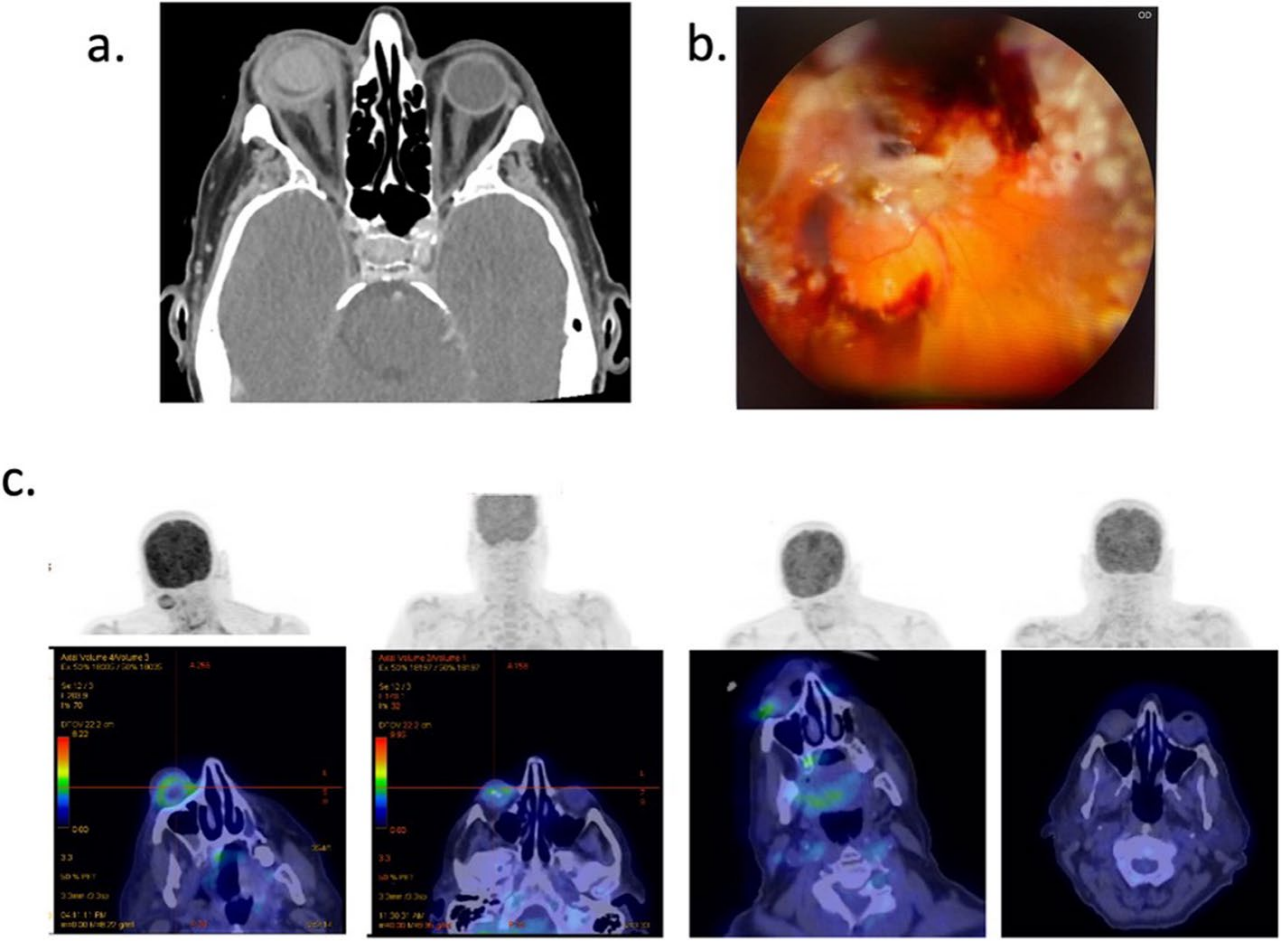
**a** Coronal computerised tomography scan of the orbit demonstrating circumferential thickening and enhancement around the entire right globe, vitreous haemorrhage, proptosis and retro-orbital intraconal inflammatory stranding. Normal CT appearance of the left globe. **B** Intraoperative right fundus photo of the right eye demonstrating large temporal retinal detachment, vitreous haemorrhage, superior area of retinitis and subretinal abscess extending inside superior arcade into macula. **C** Serial Fludeoxyglucose (FDG) positron emission tomography (PET) scans demonstrating FDG uptake and inflammatory changes in the right globe and subsequent interval improvement in right globe avidity. Images were obtained at approximately 1 month intervals from initial presentation

Right combined pars plana vitrectomy, lensectomy with insertion of silicone oil and intravitreous injection of voriconazole (100µg/0.1mL) and caspofungin (100 µg/0.1mL) was performed three days later. Intraoperative findings noted a large temporal retinal detachment and a superior subretinal abscess extending into the macula (Fig. 1b).

The patient was commenced on a loading dose of Olorofim (150mg daily) and 90mg twice daily ongoing. All other systemic antifungal therapy (voriconazole, terbinafine, micafungin) was ceased due to concern for potential drug antagonism.

However, he subsequently developed painful right ophthalmoplegia, proptosis and haemorrhagic chemosis with severe raised intraocular pressure (IOP) of 50mmHg. A positron emission tomography (PET) scan revealed disseminated disease with uptake in the right eye and the aortic valve, suggestive of fungal endocarditis. Oral voriconazole and terbinafine was recommenced in conjunction to Olorofim. Granulocyte colony stimulating factor (G-CSF) was administered for ongoing neutropenia.

The patient’s symptoms and ocular signs significantly improved whilst on triple antifungal therapy with two further doses of intravitreal voriconazole (100µg/0.1mL) one week apart. He was discharged home after a total of six-weeks as an inpatient during which he received a total of six intravitreal voriconazole injections. Oral voriconazole and terbinafine were ceased five days after discharge however the patient remained on Olorofim 90mg twice daily.

Two months post discharge, the patient was readmitted with whole body myalgias, blurred left vision, photophobia, and new left eye floaters. Left visual acuity was 6/12 which rapidly deteriorated to hand movements within 48 hours. IOP was 9mmHg. There was 2-3+ anterior chamber cells and nearly 360-degree posterior synechiae with a hazy view to the posterior segment. Both left vitreous aspiration and blood cultures grew *Lomentospora prolificans.* Intravitreal voriconazole was administered in conjunction with systemic voriconazole, terbinafine and Olorofim. The patient underwent urgent left eye pars plana vitrectomy which revealed two areas of retinitis inferiorly with a pale optic disc.

Despite intravitreal voriconazole (100µg/0.1mL), caspofungin (100µg/0.1mL) and amphotericin B (10μg/0.1mL) and systemic triple therapy, the patient died fourteen days later from severe aortic valve and femoral artery involvement of disseminated *Lomentospora*.

## Discussion

Antifungal resistance has been an increasing concern in recent years. Amongst fungal species, the emerging isolates demonstrating multidrug or inherent resistance are particularly concerning due to lack of treatment options and thus increased mortality rates [1].

*Lomentospora prolificans*, previously known as *Scedosporium prolificans*, is one such pathogen that is intrinsically resistant to conventional antifungals. Lomentospora is an opportunistic pathogen present in a range of environmental sources such as soil, sewage, polluted waters, and other decaying matter. The species has been detected in Australia, Europe and USA with a prominence in dry climates [1, 2]. *L. prolificans* predominantly affects immunocompromised hosts, particularly those with solid organ transplants, stem cell transplants or haematological malignancy [3].

*Lomentospora* most commonly disseminates through the bloodstream. Once disseminated, the mortality rate approaches 90% with a median time to death of 9 days [4, 5]. Rates of ocular involvement are estimated to be approximately 20% in the limited number of reported cases [6].

Olorofim (formerly, F901318) is a novel agent and the first drug of the new orotomide class of antifungals. Its novel mechanism of action inhibits fungal dihyroorotate dehydrogenase (DHODH) enzymes involved in pyrimidine synthesis which results in a loss of essential substrates for cell wall integrity and DNA replication leading to cell lysis [7]. Olorofim has an oral bioavailability of up to 82%, is highly protein bound and able to achieve therapeutic concentrations in the liver, kidney, lung and brain [7].

Due to its novel mechanism of action, Olorofim may be useful for fungal species that are otherwise resistant to other treatments such as azole-resistant Aspergillosis, Scedosporiosis, Lomentosporiosis and other less common mould infections [8]. Olorofim is currently in a Phase 2b open-label study (FORMULA-OLS, NCT03583164) which has enrolled 203 patients with *Lomentospora prolificans, Scedosporium spp., Aspergillus Spp*., and other resistant fungi without alternative treatment options. Preliminary results of the first 100 participants, has demonstrated 44% of participants had a complete or partial response to treatment at day 42 [9].

Previous case reports of disseminated *L.prolificans* with ocular involvement generally describe rapidly fatal disease [10]. Tio *et al*. reported a case of disseminated *L. prolificans* with ocular involvement that was successfully treated with Olorofim monotherapy. Notably, it was commenced 11 months from the initial infection [11]. In our reported case, the patient continued to deteriorate on Olorofim monotherapy however demonstrated significant improvement once voriconazole and terbinafine were recommenced in conjunction with Olorofim. Whilst it is difficult to ascertain the specific extent to which Olorofim contributed to the period of disease stability, the treatment regime appeared to be effective. His survival of 116 days from initial presentation was significantly higher than the reported median of 9 days for disseminated *L.prolificans*. Furthermore, his deterioration and left eye involvement occurred after voriconazole and terbinafine were ceased. This raises the question of whether synergistic properties between these antifungal agents exist. No effects of drug antagonism were evident when Olorofim was used in conjunction with voriconazole and terbinafine. Although other factors such as improved neutropenia from G-CSF may have also contributed during his period of disease improvement/stabilisation.

Whilst infection within the highly vascular chorioretinal layers can be treated with systemic antifungal therapy, vitritis and/or sight-threatening infection necessitates intravitreal therapy to reach therapeutic antifungal concentrations. However, the data for this approach predominantly comes from small case series [12]. Voriconazole exhibits broad antifungal activity with a favourable safety profile and good ocular penetration. Additionally, it demonstrates activity against amphotericin B-resistant *Aspergillus, Fusarium*, and other species. Due to the paucity of new cases of fungal endophthalmitis, large randomised controlled studies have not been feasible. A systematic review by Xie *et al.* found non-standardised treatment regimes in reported cases of fungal endophthalmitis where intravitreal voriconazole was recommended as initial empiric therapy in suspected fungal endophthalmitis [13].

Dual intravitreal antifungal therapy with voriconazole and caspofungin or amphotericin B has been described in the literature however its efficacy compared to monotherapy cannot be assessed due to limited cases [12]. Despite the inherent resistance of *Lomentospora* to voriconazole, our case demonstrated improvement in the right eye during intravitreal voriconazole as monotherapy. Furthermore, Chiam *et al*. reported a case of *Lomentospora* in a 9-year-old girl successfully treated with intravitreal voriconazole with systemic voriconazole terbinafine and caspofungin. Although notably in Chiam’s case the patient’s immune function recovered during treatment [14].

## Conclusion

We report a rare case of disseminated *Lomentospor*o*sis* with panophthalmitis in an immunocompromised host initially responsive to systemic Olorofim, voriconazole and terbinafine in conjunction with pars plana vitrectomy and intravitreal voriconazole. Despite presumed inherent resistance, intravitreal and systemic voriconazole appeared to limit disease progression in the right eye. Given the period of rapid improvement and prolonged survival prior to subsequent deterioration, our case demonstrates the potential of Olorofim in treating disseminated *L. prolificans* without any obvious drug antagonism with voriconazole or terbinafine when used as triple therapy. The potential synergistic effects of combined antifungal therapy with orotomides warrant further investigation.

## Data Availability

No datasets were generated or analysed during the current study.
